# Mercury in precipitation over the coastal zone of the southern Baltic Sea, Poland

**DOI:** 10.1007/s11356-014-3537-9

**Published:** 2014-09-06

**Authors:** Patrycja Siudek, Lucyna Falkowska, Aleksandra Brodecka, Artur Kowalski, Marcin Frankowski, Jerzy Siepak

**Affiliations:** 1 Department of Marine Chemistry and Environmental Protection, Institute of Oceanography, Gdansk University, Marszalka Pilsudskiego 46, 81-378 Gdynia, Poland; 2 Department of Water and Soil Analysis, Faculty of Chemistry, Adam Mickiewicz University in Poznan, Umultowska 89b, 61-614 Poznan, Poland

**Keywords:** Hg, Wet deposition, Anthropogenic emission, Rainfall, Urban atmosphere

## Abstract

An investigation of atmospheric mercury was conducted in the urban coastal zone of the Gulf of Gdansk (Baltic Sea, Poland) in 2008. Rainwater samples were collected in bulk samplers and Hg concentration was determined using AAS method. Total mercury concentration ranged from 1.9 to 14.8 ng l^−1^ (the mean was 8.3 ng l^−1^ with standard deviation ±3.7), out of which about 34 % were water-soluble Hg(II) forms. Distribution of Hg species in rainwater was related to both the emission source and the atmospheric processes. During the sampling period, two maxima of Hg concentration in precipitation were observed: the first in the cold season and the second one in the warm season. Elevated concentrations of Hg in wintertime precipitation were generally the result of local urban atmospheric emission connected with the following anthropogenic sources: intensive combustion of fossil fuels in domestic furnaces, individual power/heat generating plants, and motor vehicles. During summertime, Hg° re-emitted from contaminated land and sea surfaces was photochemically oxidized by active atmospheric substances (e.g., hydroxyl radicals, hydrogen peroxide, halogens) and could be an additional source of atmospherically deposited Hg. The results presented in this work indicate that rainwater Hg concentration and deposition values are not much higher in comparison with other urban locations along the Baltic Sea basin and other coastal cities. However, the elevated mercury concentration in rainwater and, consequently, higher deposition ratio could appear occasionally as an effect of intensive anthropogenic emissions (domestic heating) and/or photochemical reactions.

## Introduction

Atmospheric mercury occurs predominantly in the inorganic form, including: (i) gaseous insoluble elemental Hg° (GEM), (ii) less volatile and highly water-soluble reactive gaseous Hg species (GOM, e.g., HgO, HgCl_2_, HgBr_2_, Hg (OH)_2_), and (iii) total particulate Hg (TPM), with a minor role of organic compounds (e.g., monomethylmercury, other complexes). Among all the processes which are of crucial importance to mercury transport and transformation in the troposphere, particular attention should be given to the deposition mechanism that links aquatic/terrestrial and atmosphere systems.

Mercury in rainwater occurs mainly as reactive-oxidized (RGM) and particulate (TPM) species which are associated with soot, ashes, and other airborne compounds (Lindberg and Stratton 1998). A group of meteorological factors that influence chemical reactions of mercury in the atmosphere includes the following parameters: air temperature, atmospheric pressure and relative humidity, solar radiation, wind speed and direction, mixing height, thermal inversion layers, advection, long-range transport, turbulent diffusion, and stagnation and recirculation conditions as well as tropopause properties (height of marine and planetary boundary layers). The type and time of precipitation are also important factors. When considering mercury speciation in rainwater, the chemical composition and pH of rain/cloud droplets, dissolved/suspended organic matter, and acidic compounds which catalyze Hg oxidation/reduction reactions affect Hg aerosol dissolution (Munthe and McElroy 1992). Recent studies have demonstrated that low pH of precipitation creates conditions in which methylation of Hg^2+^ into its most toxic derivatives occurs (Hammerschmidt et al. 2007).

In order to estimate the contribution of anthropogenic sources to the biogeochemical cycling of toxic mercury species in the coastal ecosystem, the atmospheric measurements of these Hg forms was applied. The purpose of this research was to determine mercury concentration and atmospheric fluxes on the basis of Hg quantitative analysis in various types of precipitation: rain, snow, and mixed rain with snow. The seasonal variability and distribution of Hg were examined in relation to different meteorological factors and the proximity of both natural (marine and land) and anthropogenic sources. This work is based on the first long-term research project on atmospheric mercury transformation in the coastal area of the southern Baltic Sea with a significant urban emission, and provides an important chemical data for transport modelling, air quality, and marine environmental protection strategy. We also wished to verify the HELCOM calculations which indicate that Poland is responsible for the largest input of mercury to the Baltic Sea.

## Material and methods

### Description of the measurement site

**Fig. 1:**
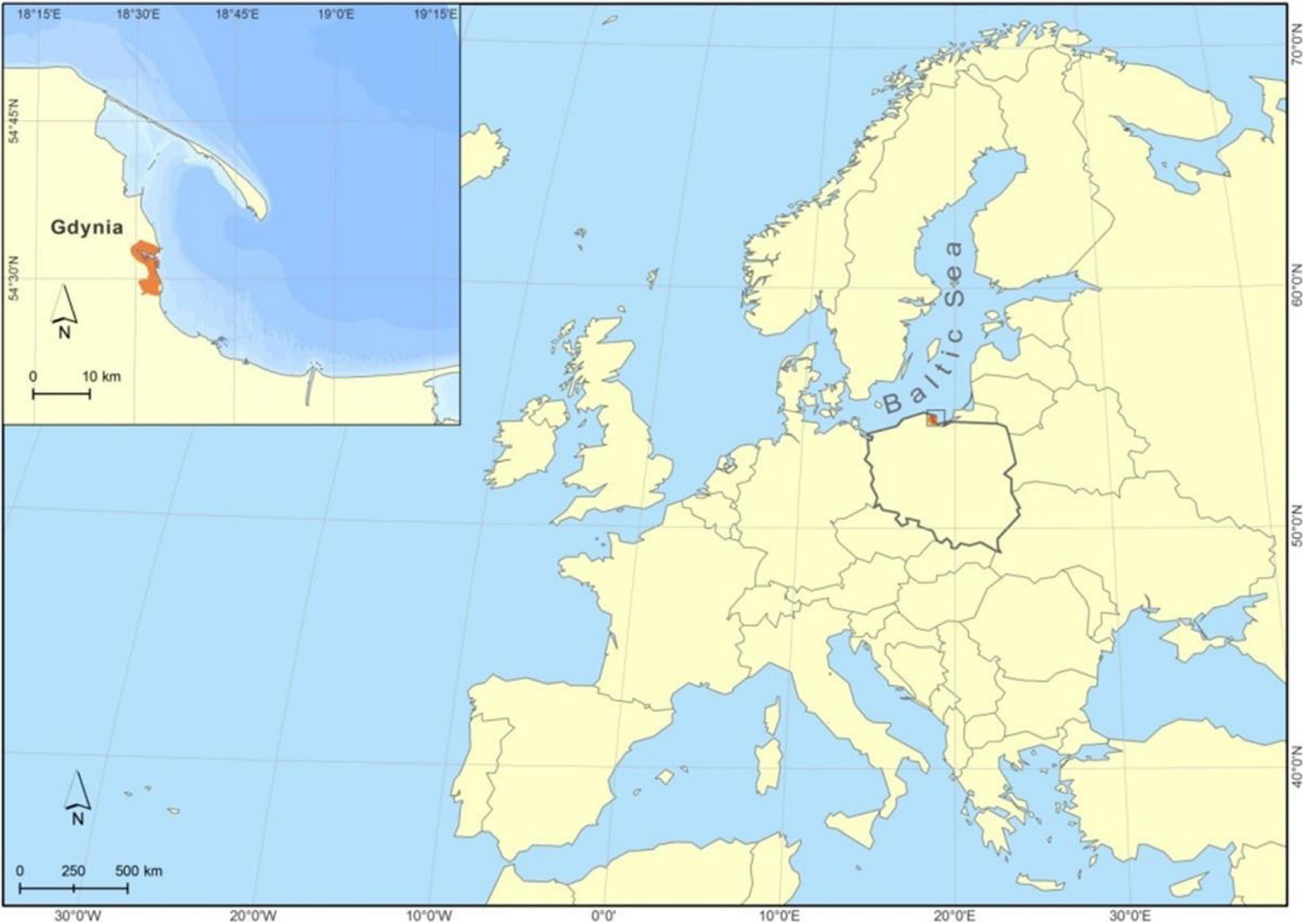
Sampling location for Hg measurements in Gdynia (Poland) between January and December 2008

Rainwater samples were collected in Gdynia during the entire year of field measurements. The sampling site (*φ* = 54°30′34″N, *λ* = 18°32′30″E) was representative for the surrounding urban Pomerania region, situated ∼100 m from the heavy-traffic road and about 1,000 m from the coastline, on the roof of the Oceanography Institute of Gdansk University (20 m above ground level and above the tree-tops) (Fig. 1).

Gdynia is located in an industrial area of the Polish coast of the Baltic Sea. The sampling location is under the constant influence of different inland (regional and local) and marine emission sources. The most important industrial and urban activities in Pomerania region and nearby area are the following: coal-burning heat and power plants, heat-generating stations, and domestic furnaces, where coal is the main fossil fuel. Moreover, such sources as, dumping grounds for municipal wastes, hospital and domestic sewage, cement factories, and sewage treatment plants as well as different industrial units producing metals and paints, chemical industry, the refinery, docks, and shipyards, also contribute to the Hg emission. All the mentioned anthropogenic mercury sources are situated within 50 km from the sampling site. In this study, we used term “heating season” to highlight the special period during wintertime when intensive domestic heating occurs. The whole heating season is about 5–6 months of the calendar year, typically from the midst of October to the end of March.

### Hg sampling

Rainwater samples for total and water-soluble Hg (operationally defined as divalent Hg) analysis were collected during continuous measurement series at the coastal sampling site in 2008. The bulk sampler consisted of a Teflon funnel (reception surface = 0.0314 m^2^) connected by a Teflon-lined tubing with an acid-cleaned borosilicate glass bottle. Samples were collected during precipitation or straight after it to prevent negative artifacts associated with background contamination, Hg adsorption on sampler walls, etc. Bottles used for rainwater collection were rigorously prepared using standard cleaning procedure: rinsing with double deionized water (DDW), soaking with 10 % nitric acid for 48 h, rinsing with DDW five times, drying in a laminar flow bench packed with high-efficiency particulate air (HEPA) filters (Alpina K700), and storing in zip-locked bags. After each rain episode, sampling set was cleaned with DDW and replaced with a new plastic bottle, while the collected samples were transported to the laboratory, and then pre-acidified using HCl (Sigma Aldrich) in the amount of 0.5 % per volume in order to eliminate Hg loss and stabilize pH to the value of <2. All samples were protected from light and stored at 4 °C in borosilicate glass bottles prior to the main analysis. Snowfall samples were melted thoroughly at room temperature. To assure high-quality data and estimate uncertainties for the collecting system, the sampling procedure, storage period, and field blank values were controlled monthly. The tests of the abovementioned procedures (transport, calibration, preservation) confirmed no contamination of the samples during system handling. The blank values (bottles rinsed with DDW) were under the detection limit of the analyzer and showed no contamination when rain sampling. The values for background samples were not higher than 10 % of Hg concentration measured for individual cases. The precipitation amount (in mm) was measured manually using a Hellman rain gauge.

### Mercury measurements and QC/QA procedure

Mercury concentration in rainwater samples was determined through the use of atomic absorption technique, after the oxidation by BrCl and the reduction by stannous chloride (3 % SnCl_2_) to Hg°. According to the US EPA Method 1631, the excess BrCl was immediately neutralized by addition of a relevant amount of hydroxylamine hydrochloride (NH_2_OH · HCl). Total and soluble mercury concentration in unfiltered snow samples (THg) was quantitatively determined using a Gardis-3 mercury analyzer (given values are not volume-weighted mean concentrations). A detailed description of that instrument can be found elsewhere (Urba et al. 1995). The analytical method is based on gold amalgamation and atomic absorption detection.

In order to determine the precision and accuracy of the analytical procedure, a five-point calibration curve was performed. The percentage recovery of Hg from duplicate spike standard solutions measured at the beginning and every ten samples ranged from 98 to 103 %. Additionally, in order to verify and control the applied method, certified reference material was used (ORMS-3, National Research Council Canada). Method recovery was on the level of 98 ± 5 %. The detection limit of the analytical method, based on three times the standard deviation of blank samples, was 1.0 ng l^−1^. Standard solutions and reagents were ultrapure and of high-quality grade (Merck). Analyses were carried out in an ultraclean laboratory meeting the standards specified for trace metals analyses (particle-free gloves, protective clothing, laminar flow chamber), where Hg concentration in the air was relatively low. The average bottle blank value obtained by measuring Hg concentration in ultrapure Milli-Q water (18.2 M Ω cm^−1^), which was used to clean the PTFE reception surface, was <0.5 ng l^−1^. In this article, we use the following abbreviations: THg (total mercury) and Hg^2+^ (reactive, water-soluble Hg measured by direct reduction of Hg^2+^ to elemental Hg° in SnCl_2_ solution).

### Meteorological parameters

Environmental conditions for each sampling period were described on the basis of primary meteorological factors, i.e., temperature, humidity, pressure, direction, and velocity of wind (10-s record averaged to 30 min). Meteorological data were recorded continuously by the Huger Weather Station. Backward trajectories (BTs) of air masses for each rain episode were retrieved from the NOAA Hybrid Single-Particle Lagrangian Integrated Trajectory model (HYSPLIT, Draxler and Rophl 2003), based on GDAS global meteorological data (archive, 2005 to present). The trajectories were initialized every 6 h (at 0:00 am, 6:00 am, 12:00 pm, and 6:00 pm) at starting heights 500 and 1,000 m (to identify the potential Hg source regions during the long-range air parcel transport towards the sampling location) above Gdynia site. Two last variables correspond to the upper threshold of marine boundary layer (MBL), respectively, in dark and light conditions, and the run time for trajectories reflects the typical atmospheric residence time of particulate mercury in fine particles. The total number of 132 trajectories was calculated. They were then attributed to one of the separated sectors:

(i) Maritime (air masses from NW-N directions, representing clean marine air with Hg values of biogeochemical background and minor contribution of anthropogenic sources)
(ii) Mixed maritime-continental (air masses from NNE-E, air with relatively high pollutants content)
(iii) Continental (W-SW and SSW-S-ESE, mostly very polluted air masses, originating from industrially developed regions)

The attribution of air masses was done visually under the condition that at least 50 % of the air mass was included in the area designated by the borders of the sector (the division along the 54th parallel).

## Results and discussion

### Mercury concentration in precipitation and its comparison with other coastal sites

The statistical summaries for total THg and divalent Hg^2+^ concentrations in rainwater samples of the urban coastal area in the southern Baltic Sea has been depicted in Table 1.

**Table 1:** Total and reactive water-soluble Hg species (ng l^−1^) in precipitation over the urbanized coastal zone of the Gulf of Gdansk in 2008, Poland

Statistics of data	Hg^2+^	THg
Mean	2.6	8.3
Median	1.9	7.7
Minimum	0.8	1.9
Maximum	10.9	14.8
Lower quartile	1.2	5.0
Upper quartile	3.4	11.7
5 % percentile	0.9	2.9
95 % percentile	6.0	14.2
Standard deviation	2.0	3.7

The average THg concentration in precipitation measured in 2008 was 8.3 ng l^−1^ (SD = 3.7). The total mercury content in the collected samples ranged between 1.9 and 14.8 ng l^−1^, whereas 90 % of values were within the range of 2.9–14.2 ng l^−1^. The concentration of divalent Hg^2+^, operationally defined as reactive mercury species (easily reducible Hg) oscillated between 0.8 and 10.9 ng l^−1^, constituting on average 6–94 % of the total mercury (Table 1). The mean Hg(II) level during the whole sampling period was 2.6 ng l^−1^, with a standard deviation = 2.0 ng l^−1^.

**Fig. 2:**
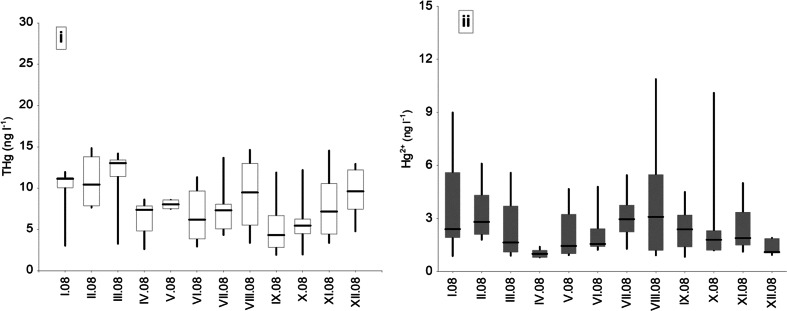
Variability in (*i*) THg and (*ii*) Hg^2+^ concentration (ng l^−1^) in precipitation over the city of Gdynia in 2008 (*box* upper and lower quartile and *whisker* minimum and maximum plots for mercury concentrations)

During the sampling period in 2008, total and divalent Hg concentrations in rainwater samples showed significant differences (Kruskal-Wallis nonparametric test, THg *p* value = 0.0215, Hg^2+^ *p* value = 0.0165). Figure 2 shows seasonal pattern of THg and Hg^2+^ in precipitation collected in the following seasons: spring (March to May, *n* = 24), summer (June to August, *n* = 44), fall (September to November, *n* = 36), and winter (December to February, *n* = 28). In general, averaged rainwater Hg concentrations for the both selected compounds had slightly different seasonal distribution (Fig. 2, Hg^2+^/THg, *R* ^2^ = 0.0599, Mann Whitney *U* test, *p* < 0.001). It was found that atmospheric mercury level in liquid phase changed within seasons as a result of emission and meteorological factors.

The highest monthly total Hg concentrations (over 10.0 ng l^−1^) were reported either in the middle of the heating season (January to February) or at the end of winter (March) during large-scale and convectional precipitation events (Fig. 2i). At the beginning of spring (April), the average value for mercury concentration in rainwater was relatively low (mean = 7.0 ng l^−1^) until the warmer days of the vegetation season came (June to August), when the concentration of Hg in precipitation started to become higher. During summertime, the increase of mercury content in precipitation (mainly as Hg^2+^ and particulate-phase Hg) can be explained by some natural processes, i.e., release of Hg° into the atmosphere not only from the surrounding contaminated areas (soils, vegetation) but also from marine systems. Similar cause-effect relationships that generate local short-term increasing of atmospheric Hg level during the warm season were observed at other locations (Marks and Beldowska 2001; Wängberg et al. 2008). Additionally, it was found that elevated Hg concentration in rainwater during the season of intensive sun radiation and high temperature could be the result of atmospheric oxidation of gaseous elemental mercury to the highly reactive forms (Hg^2+^ or Hg^1+^) in the presence of OH radicals, halogens (Engle et al. 2008). Moreover, a large variability of meteorological conditions (i.e., turbulent diffusion, land-sea breeze) and the altitude of marine boundary layer could also affect diurnal and seasonal fluctuations of atmospheric Hg in various chemical form (e.g., gaseous, aqueous, and/or particulate) and modify its biogeochemical cycling in coastal environments. In the present study, total mercury concentrations in precipitation over the coastal zone of the southern Baltic in autumn (September to October) were lower again as compared to spring and summer (Fig. 2i).

Similarly to the total Hg values, soluble inorganic fraction (Hg^2+^) demonstrated seasonal variability and relatively high range of concentrations over the study period (Fig. 2ii). We found that time-related data of Hg^2+^ had much more clear bimodal distribution than total Hg. During the sampling period in 2008, two peaks of Hg^2+^ species in liquid phase were observed, first in winter (January 9.0 ng l^−1^) and second in summer (August 10.9 ng l^−1^). Over the coastal zone of the southern Baltic, concentrations of divalent mercury in rainwater samples were, on average, four times lower compared to total Hg. The lowest seasonal average Hg(II) concentrations in precipitation were observed in spring (April 1.0 ng l^−1^) and winter (December 1.3 ng l^−1^), when the average amounts of rainwater were 49.2 and 30.0 mm, respectively. The highest averaged levels of Hg species in precipitation were reported in February and August, and were calculated to be 3.0 ng l^−1^. For winter precipitation, apart from the major industrial/combustion emission sources, the second important factor causing the increase of Hg can be chemical transformation, i.e., condensation of gaseous Hg-enriched compounds onto aerosol surface at lower temperatures, since they can be rapidly conversed into much easier deposited species (Amos et al. 2012). During summertime, an important process maximizing the amount of Hg fraction in precipitation seemed to be Hg evasion from soil and sea surface and the contribution of photochemical Hg° oxidation. Moreover, many authors concluded that an extremely high level of particle-bound Hg in the air over the urban areas clearly coincide with elevated concentration of other anthropogenic pollutants (e.g., O_3_, SO_2_, PAHs, BTX, radicals). These interactions are particularly important for coastal environments due to the high risk of air, water, and soil contamination.

It was found that Hg^2+^ variability in precipitation over the urbanized costal environment of the southern Baltic Sea is probably caused by the same reason as for total Hg (comparable shape of the seasonal pattern). However, especially during summertime, the dominant role of the sea as a source and sink of atmospheric mercury was revealed. Similar influence of marine emission and meteorological conditions was highlighted in several works (Ci et al. 2011).

Recent studies emphasized a wide variability in concentration and deposition rate of speciated mercury in the atmosphere which depends on location and sampling period, including proximity of various anthropogenic and natural emission sources, photochemical reactions, synoptic situation, and aerosol processes (Ebinghaus et al. 1995; Engle et al. 2008; Wängberg et al. 2008). Data in Table 2 indicate differences in Hg concentration levels between the individual locations classified as rural, urban, background, and industrial. The concentration range of mercury in precipitation over the coastal zone of the southern Baltic was much lower compared to the values measured at the Baltic EMEP stations (Zingst, Germany). The mean Hg level in precipitation samples collected within short-term measurements in 1995 at the urban site of the Florida Mercury Atmospheric Study (FAMS) did not exceed 20 ng l^−1^, with the highest value of about 82 ng l^−1^ reported during the warmer season (Landing et al. 1998). Our results contrasted with some highly polluted rural (Guo et al. 2008) and urban areas (Fang et al. 2004) in China, where the extremely high Hg concentrations in precipitation (∼149.1 and ∼718.0 ng l^−1^, respectively) were determined.

**Table 2:** Comparison of total Hg concentration (ng l^−1^) in precipitation

Study area	Site classification	Sampling period	THg	Reference
Gdynia, Poland	Urban	2008	1.0–14.8	This study
Zingst, Germany	Marine	1998–1999	20.0–110.6	HELCOM (2006)
Preila, Lithuania	Marine/rural	2005–2006	10.0–30.0	Milukaite et al. (2008)
Wujiang, China	Rural	2006	7.5–149.1	Guo et al. (2008)
Changchun, China	Urban	1999–2001	150.0–718.0	Fang et al. (2004)
Davie, U.S.	Urban	1995	4.0–82.0	Landing et al. (1998)
Mace Head, Ireland	Background	1992	6.1–37.8	Ebinghaus et al. (1995)
Cabo de Creus, Spain	Industrial	2003–2004	23.0	Wängberg et al. (2008)

The range of mercury concentration in precipitation samples from Gdynia was comparable to the results from the Spanish sites along the Mediterranean Basin, with a mean value of 23 ng l^−1^ (Wängberg et al. 2008). Levels of Hg in precipitation at Gdynia sampling site were slightly lower than those observed at Preila (eastern Baltic, the Lithuanian coast) by Milukaite et al. (2008). We used different sampling periods in the abovementioned comparison; thus, the results given do not reflect the actual situation thoroughly, but we hope to highlight the visible changes in rainwater Hg concentration (mostly downward trend) that reflect its complex transformation pathway in the atmosphere, strongly associated with the features of the sampling location.

### Mercury in urban snow

Rain (*R*), snow (*S*), and mixed precipitation samples (*M*) were collected during cold season in 2008 (January to March and November to December), when local and regional combustion of fossil fuel strongly influenced atmospheric Hg chemistry, transport, and deposition processes. Most studies concerning chemical and physical properties of Hg in snow and ice samples are based on boreal and polar observations (Dommergue et al. 2003; Douglas et al. 2008). In the present study, focused on the region where snow cover occurs seasonally, we introduced sample type categorization (*S*, *R*, *M*) which may be helpful while explaining differences in Hg chemistry. By using the division based on the type of precipitation, we could identify Hg variability and distribution pattern during the specific sampling period controlled predominately by air temperatures below 0 °C and low solar radiation. It seems that Hg-scavenging mechanism (*S*, *R*, *M*) plays an important role in mercury distribution during wintertime. Therefore, such approach would simplify studying the relationships between type of precipitation and Hg concentration.

During measurements conducted in winter over the urban coastal area of the southern Baltic Sea, total and divalent mercury concentrations in rainwater, snow, and mixed samples were significantly different. It was statistically proved by Kruskal-Wallis test (*p* < 0.05). Table 3 summarizes the results of Hg deposition and THg, Hg^2+^ concentrations.

**Table 3:** Hg^2+^ and THg concentration (ng l^−1^) and deposition values (μg m^−2^) in different types of precipitation during cold sampling season in 2008 (Gdynia)

Type of precipitation	Estimator	Number	Hg^2+^	THg	Hg deposition
Snow (*S*)	*X* ± SD min-max	18	3.4 ± 1.6 1.1–5.6	9.8 ± 4.4 3.4–14.8	0.019 ± 0.043 0.003–0.180
Rain (*R*)	*X* ± SD min-max	37	2.7 ± 2.2 0.9–9.0	9.9 ± 3.0 3.0–14.4	0.050 ± 0.084 0.009–0.437
Mixed (*M*) (snow/rain)	*X* ± SD min-max	5	1.4 ± 1.0 0.5–2.9	7.1 ± 5.8 0.8–12.4	0.053 ± 0.073 0.001–0.151

Among all the considered types of precipitation, *R* and *S* events showed relatively high level of Hg as compared to the mixture of rain and snow samples. The THg average concentration levels for urban rainwater and snow samples were 9.9 ± 3.0 and 9.8 ± 4.4 ng l^−1^, respectively, while the amount of Hg found in mixed falls was 7.1 ± 5.8 ng l^−1^. Divalent Hg compounds demonstrated a different trend. Concentrations of Hg(II) in snow samples were slightly higher than in rain samples (3.4 and 2.7 ng l^−1^, respectively), and approximately two times higher than in mixed precipitation.

These results suggest that snowfalls were more enriched with reactive gaseous mercury species and particulate-bound Hg forms (via sorption) of anthropogenic origin than rain droplets or mixed precipitation. On the other hand, large variability in mean deposition values for *S*, *R*, and *M* events (0.019, 0.050, and 0.053 μg m^−2^, respectively) indicates that snow was not as effective in Hg removal from the ambient air as rain or mixed precipitation. Previous measurements also reported significant differences in Hg concentration in the rain, snow, and mixed samples, with higher deposition values related to rain (Lai et al. 2007). Results from other locations at temperate latitudes suggest that snow surface is less effective in Hg-enriched aerosol uptake than rain droplets (Landis et al. 2002), but still remains an important medium for Hg transformation and should be considered while estimating local, regional, and global budget. The laboratory studies on Hg fate and dynamics in snow structure performed by Mann et al. (2011) showed that changes in air temperature during snow cover formation are a key parameter in Hg transformation. Other studies demonstrate that anthropogenic Hg-enriched particle formation during winter time is dependent on low temperature, as a consequence of gas-particle partitioning (Amos et al. 2012).

The variability in THg concentration determined in the present study for different types of precipitation was consistent with other field measurements at urban locations in the USA (Fitzgerald et al. 1991). Ranges of Hg concentrations in snow and rain samples measured by Lai et al. (2007) were similar to our results. The researchers also observed that rainwater and mixed fall samples collected away from anthropogenic Hg sources had slightly lower content of total mercury (5.9 and 4.3 ng l^−1^, respectively), showing clear dependence between mercury concentration and a type of fall during the field experiment (Lai et al. 2007). Averaged volume-weighted concentration of mercury in snow samples during their measurements was calculated to be 4.0 ng l^−1^, despite the fact that the highest mercury concentration (51.4 ng l^−1^) was reported during the snow episode.

Since Hg transformations in the cold ambient air are affected by numerous factors, i.e., synoptic situation, rapid changes in meteorological conditions (advection, turbulent diffusion, stagnation, recirculation), type of wet (S, R, M) and dry (gases, particles) removal, and anthropogenic factors as well as chemical transformation, the Hg speciation and distribution during wintertime may vary widely. In our studies, the maximum Hg deposition flux value was observed for rain. It should be noted that in the present study, due to a low number of mixed precipitation events (*n* = 5), the result of deposition for this type of falls may be underestimated. Hence, the investigations in this field should be continued and supplemented with other chemical and physical aspects of Hg transformation, in order to fully support the given hypothesis and better understand the properties of mercury in different types of precipitation.

### Identification of mercury sources

**Fig. 3:**
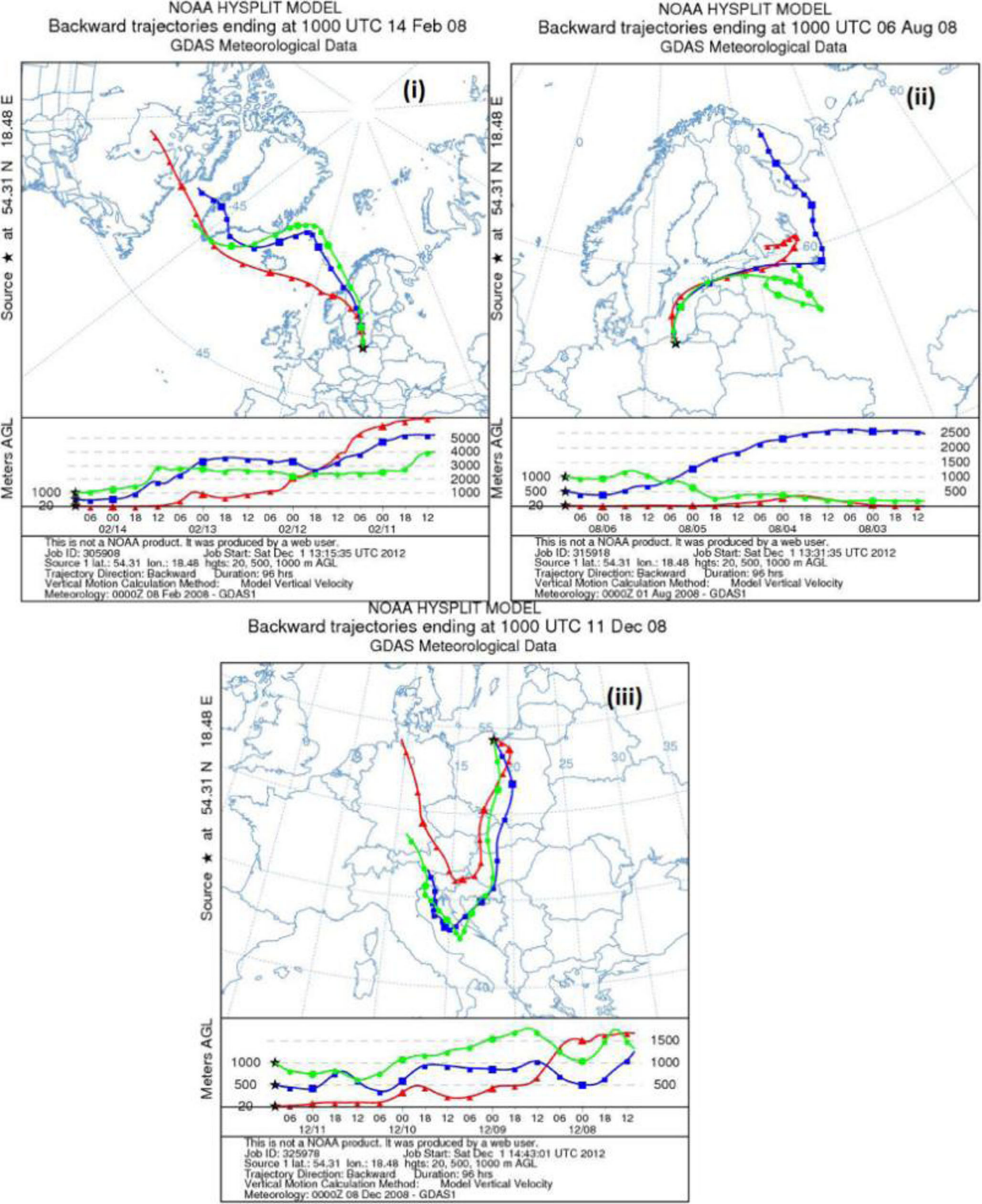
Examples of backward air mass trajectories for the selected sectors (*i*) maritime, (*ii*) mixed, and (*iii*) continental associated with elevated Hg concentration in precipitation over the southern Baltic Sea during the study period

It is well known that intensive solar radiation and high concentration of oxidants (i.e., ozone, hydrogen peroxide, nitrogen, and hydroxyl radicals) influence mercury transformation in the atmosphere (Selin et al. 2007). Spatial and temporal fluctuations of Hg compounds, including Hg° and particulate-bound and water-soluble species, are controlled not only by the emission range but also by airborne transport and dispersion conditions. Studies on chemical composition of aerosols over the Baltic Sea proved that air masses specific for this region should be examined in detail (Siudek et al. 2011). The similar approach was applied in the present study. It was based on the analysis of 96-h backward trajectory maps. In Fig. 3, we demonstrated the examples of bulk rainwater mercury concentration in samples assigned to the selected air mass classes.

It is important to point out that local meteorological conditions during the sampling period and the backward trajectory analysis (showing the impact of distant Hg sources) were studied for all cases, but in the present paper, we discuss only those events and parameters (in particular, wind speed, and direction) that were surely related to elevated concentrations of Hg in precipitation.

During sampling period, the variations of Hg concentration in rainwater over the urbanized coastal zone of the southern Baltic Sea were dependent on meteorological conditions controlling Hg emission and distribution processes (Fig. 3). Initially, we expected that local/regional anthropogenic emission would clearly coincide with long-range Hg transport from highly polluted urban/industrial regions and finally cause the enhanced Hg concentration in precipitation. Interestingly, the maximum concentration of total Hg in precipitation (14.8 ng l^−1^) was reported in wintertime during the Atlantic circulation when air masses were arriving from the marine sector (including NW-N-NNE wind direction, Fig. 3i). Elevated Hg concentrations in rainwater were primarily associated with local and regional coal-burning sources as well as ship traffic and harbor activities. That could be supported by the fact that before the precipitation, we observed local stagnation, suggesting that a large fraction of Hg measured in liquid phase was of local anthropogenic origin. Low velocity of wind (<1 m/s) and its prevailing direction from the maritime/mixed sectors create the conditions under which the Hg accumulation over the sampling site can occur. Additionally, BT plot for 14/01/08 rain episode showed that Hg could be transported from remote northern locations in Greenland and Canada (Fig. 3i).

In contrast, during summertime, we identified the event of high Hg concentration in rainwater (14.6 ng l^−1^, 06/08/08, Fig. 3ii) during the northern advection. Meteorological conditions associated with that event were particularly affected by air masses that had recently passed over the northern part of the Baltic Sea and the areas of Russia, indicating the dominant contribution from the sea surface and minor influence of anthropogenic emission. It should be noted that marine sector (especially open sea water) additionally includes the unknown contribution from shipping emission (ship plumes) that might be of crucial importance for the estimation of atmospheric Hg budget. Moreover, in humid air masses from maritime sector (NW-N) and mixed maritime-continental sector (NNE-E), the main Hg source in precipitation was identified as atmospheric processes of Hg and halogen (sorption on marigenous particles) and local sources (harbors, shipyards, industrial activities). Based on detailed wind speed analysis, it was found that high Hg concentration in precipitation was frequently observed under low wind speed from sea (<2 m s^−1^, THg/Vw *R* ^2^ = 0.400), which suggests that these rain episodes of elevated mercury concentrations were directly associated with land-sea breeze circulation. It was reported by some authors that elemental mercury emitted from seawater to the atmosphere could be oxidized to the reactive divalent forms by chloride radicals in marine boundary layer (Laurier et al. 2003). Following these chemical reactions, reactive mercury species could be transported inland during the flow of sea breeze, affecting the increase both in the variability and range of Hg concentration in precipitation. Similar processes in marine atmospheric conditions were observed and described previously (Laurier et al. 2003; Sprovieri et al. 2010). The presence of this mechanism was also confirmed in the studies on transformations of mercury in the Baltic Sea region (Marks and Beldowska 2001). The authors pointed out that high temperature favors mercury releasing from seawater. This process can be intensified by the increase of turbulent mixing in the sea-air boundary layer.

A detailed analysis of BT simulations for rainwater attributed to the marine sector (14/01/08 and 06/08/08) allowed to elucidate the potential impact of sites within the Polish Economic Zone of the Baltic Sea where the chemical weapons (mines, bombs, wrecks containing conventional munitions) were dumped. For both the considered cases, it was observed that air parcel was transported over those areas, suggesting strong contribution to the Hg concentration level in rainwater over Gdynia. However, during wintertime, Hg° is less efficiently emitted from open sea water to the atmosphere compared to warm period. This seasonal relationship should be further verified.

Results of the air mass backward trajectory analysis showed that long-range transport from the most polluted regions in Poland and Europe can be regarded as an important factor having the influence on Hg measurements in precipitation over the southern Baltic Sea (Fig. 3iii). The HYSPLIT 96-h air mass back trajectory at 500 and 1,000 m over the model ground level in Gdynia on 11 December 2008, clearly indicated that the atmospheric transport across distant areas with a high density of anthropogenic origin strongly affected the chemical composition of precipitation at sampling location causing elevated Hg concentration (12.9 ng l^−1^). Recent studies have showed that mercury emission to the atmosphere in Poland changed significantly over the past years. The Hg emission trend analysis revealed that it decreased from 38.8 t in 1985 to 21.0 t in 2005 (Hlawiczka et al. 2006). Nevertheless, in the southern region of Poland (Silesia), 500 km from the Baltic coast, wet deposition of Hg still remains high. Zielonka and Nowak (2009) estimated that wet deposition of total Hg in that highly industrialized and urbanized region of Poland was 28.7 mg m^−2^ in 2008, out of which 20.0 mg per 1 m^2^ was ascribed to the cold season. We observed that the area of Silesia could be the significant mercury source for the atmosphere over the Baltic Sea, while southern advection of air masses occurs, resulting in the intensification of episodes with extremely high Hg concentration in precipitation. It should be noted that similar cases, but concerning the size-fractionated airborne particulate Hg, were reported several times in this region and were directly attributed to high-temperature processes at regional scale (Beldowska et al. 2007; Siudek et al. 2011).

On the basis of a thorough analysis of local meteorological conditions, we found that the processes of Hg dispersion/accumulation prior to or during the precipitation event near the measurement site were controlled by wind speed. It was found that over the coastal zone of the Baltic, when wind velocities were <1 m/s, mercury originated from the sources located close to the sampling site. Elevated mercury levels in rainwater (average >8 ng l^−1^) were observed especially for the southern advection during wintertime. In that case, the air masses were flowing from the sector where urban centers and major pollution sources, e.g., heat-and power-generating plants, industrial plants, and many other point and mobile sources of mercury are located (Tricity Agglomeration). The highest Hg concentration in precipitation was probably caused by the emission from the nearby industrial sources (i.e., oil refinery, transshipment harbor, repair shipyard, heat and power plants) as well as from distant sources located several hundred of kilometers or more away, in strongly industrialized regions of Poland and Europe (Fig. 3iii). Lower Hg concentrations in precipitation were reported in air masses of marine origin, especially when winds were blowing from north, north-east, or east at the speeds >3 m/s (Fig. 3). Another factor, possibly influencing the relationship between Hg variability and the range of concentrations in rain samples, was the height at which air masses were flowing. The lower they were flowing, the higher were the concentrations of Hg in wet precipitation. This dependence was observed for each of the considered sectors during the sampling period while comparing the backward trajectory maps (Fig. 3).

### Mercury in rainwater in the southern Baltic Sea region

Data regarding Hg concentration in precipitation in the coastal regions of the southern Baltic Sea are rather limited and poorly documented. Thus, one of the main goals of the present study was to provide newly generated information for atmospheric transport, chemistry, and deposition of mercury.

It should be highlighted that data presented here were a part of a multidisciplinary research project focused on long-term mercury transformation in the atmosphere of the southern Baltic Sea basin (Beldowska et al. 2008; Siudek et al. 2011). In Table 4, we compared Hg rainwater concentration and diurnal/annual deposition fluxes in field measurements (Ebinghaus et al. 1995; Schmolke et al. 1997) and in model computations (Boszke 2005; Petersen et al. 1995). We found that official calculations of Hg concentration in rain samples for this region (HELCOM 2006) were much higher than obtained in the present study. For example, Schmolke et al. (1997) found that Hg inflow to the Bay Puck ecosystem (inert part of the Baltic Sea) with wet precipitation (rain, snow) was the same as in the southern Baltic, 26–72 ng m^−2^ day^−1^ (Table 4). If the input from dry deposition was considered, these values proportionally increase, suggesting that dry deposition has an important input to Baltic Sea ecosystem (10 % of the total deposition, Petersen et al. 1995). The range of diurnal wet and dry deposition of Hg found by Ebinghaus et al. (1995) was slightly higher (Table 4).

**Table 4:** The comparison of Hg concentration, diurnal and annual deposition flux calculated for the sampling period of 2008 and discussed with other data

	Gdynia sampling site	Bay of Puck	Southern Baltic Sea
Hg concentration (ng l^−1^)	8.3 (1.9–14.8)^c^	–	(20–110)^b^
Diurnal wet deposition of Hg (ng m^−2^ day^−1^)	–	(26–72)^a^	–
Diurnal wet and dry deposition of Hg (ng m^−2^ day^−1^)	–	(29–80)^a^	100 (35–190)^b^
Annual deposition of Hg	4.0 (μg/m^2^)^d^	(1.1–3.0)^a^	3.8 (1.3–7.2)^b^

^a^Calculations by Boszke (2005) and Schmolke et al. (1997) in kg year^−1^

^b^Calculations by Ebinghaus et al. (1995)

^c^This work

^d^Annual deposition value in 2008 was calculated for precipitation amount of 655.6 mm year^−1^

If we spatially interpolated those values onto the whole area (i.e., Gulf of Gdansk, ca. 25 600 km^2^), the atmospherically deposited mercury would exceed 7.2 kg and would be ca. 3 times higher than the results presented for the Bay of Puck (Table 4). We suppose that uncertainties are generally associated with differences in time (changes in Hg emission), regions, and seasons (meteorology). Considering HELCOM data, it is clearly visible that Hg emission within the Baltic Sea region has decreased since 1996, even though the spatial differences in Hg concentration and deposition are still significant.

Due to the fact that the available data related to spatial distribution of mercury within the Baltic Sea region indicate that Polish coastal environment is the most polluted among all the countries belonging to the HELCOM’s community, we wanted to provide an evidence to verify those findings. Our results demonstrated that Gdynia—as a representative site of the urbanized coastal zone of the Gulf of Gdańsk—has no significantly higher mercury deposition values than other cities located at the coast of the Baltic Sea, e.g., Aspvreten (Sweden), Zingst (Germany), and Råö (Denmark). The average total mercury concentration in rainwater samples calculated for this study did not exceed 9.0 ng l^−1^ and was ca. 7 times lower compared to the values previously recognized. However, it was also observed that in some sampling periods, when a combination of special meteorological conditions (thermal inversion layer, height of atmospheric boundary layer) and elevated anthropogenic emission of particulate-bound and reactive gaseous Hg appears, extremely high rainwater mercury concentrations might occur more frequently. As noted in this study, winter season with a high emission of pollutants from domestic heating would inevitably influence chemical transformations (gas-/Hg-enriched airborne particles) and wet/dry Hg removal. This is particularly important for marine ecosystems influenced by human activities.

## Conclusions

Annual observations of mercury in rainwater samples were performed for the first time in the industrialized region of the southern Baltic Sea. Data obtained from measurements in 2008 revealed a wide variability in Hg concentration. The average rainwater concentration of the total mercury was 8.3 ± 3.7 ng l^−1^, out of which approximately 34 % constituted divalent species.

Seasonal changes in rainwater Hg concentration were influenced mainly by local and regional anthropogenic emission sources (industrial/combustion) and atmospheric conditions. We found significant differences in the occurrence of a maximum value for THg and Hg^2+^, indicating the source-related relationships for the selected compounds. Water-soluble Hg species presented bimodal character with a double peak during the entire sampling period. For both Hg species, higher levels were more often reported in wintertime precipitation, indicating that the anthropogenic emission, presumably from local and distant coal-fired power plants, was strongly enhanced and connected with the heating demand. In Poland, fossil fuels (especially hard and brown coal), are still the basic source of energy. However, data available from the national emission inventories showed a significant reduction in Hg emission of industrial origin for the last decade. In the urbanized coastal zone of the southern Baltic Sea, during warm season (spring and summer), apart from anthropogenic Hg emission sources, the additional input of mercury to the ambient air could be caused by re-emission from the seawater and surrounding contaminated environments. This phenomenon could occur more frequently in the periods of higher solar radiation and higher temperatures (possibly photochemical oxidation of gaseous Hg).

Apart from local anthropogenic emission sources, which introduced mercury to the coastal air, an important influence on the high concentration of this metal in precipitation had the type of circulation with prevailing air mass advection from the most polluted regions of Poland (Silesia) and other countries (Russia, Ukraine, Belarus, Germany, Czech Republic, Slovakia, Austria). An additional parameter which also controlled the amount of Hg in precipitation was the height of the airflow. The lower the air mass was flowing, the higher was the concentration of Hg in wet precipitation. This dependence was observed during all the selected seasons.

Mercury concentrations in rainwater samples from the present study are comparable with the results from other coastal regions of the Baltic Sea. Therefore, data available in official reports concerning deposition of Hg to the coastal area of Poland should be verified.
